# Dual‐tDCS combined with sensorimotor training promotes upper limb function in subacute stroke patients: A randomized, double‐blinded, sham‐controlled study

**DOI:** 10.1111/cns.14530

**Published:** 2023-11-23

**Authors:** Chong Li, Yun Chen, Shuting Tu, Jiaying Lin, Yifang Lin, Shuo Xu, Maohou Wu, Yong Xie, Jie Jia

**Affiliations:** ^1^ Department of Rehabilitation Medicine First Affiliated Hospital of Fujian Medical University Fujian China; ^2^ Fujian Branch of Huashan Hospital Fudan University Fujian China; ^3^ National Clinical Research Center for Aging and Medicine, Huashan Hospital Fudan University Shanghai China

**Keywords:** fNIRS, rehabilitation, sensorimotor training, subacute stroke, tDCS

## Abstract

**Background:**

Dual transcranial direct current stimulation (tDCS) over the bilateral primary somatosensory cortex (PSC) has potential benefits in stroke. In addition, compared with traditional rehabilitation training, sensorimotor training can significantly improve the sensorimotor function of patients. However, the efficacy of dual‐tDCS combined with sensorimotor training in patients with subacute stroke is unknown.

**Objective:**

To assess whether dual‐tDCS may enhance the efficacy of sensorimotor training on the upper limb functions in patients with subacute stroke. In addition, this study aims to explore the potential clinical mechanism of this combination therapy.

**Methods:**

We randomized 52 individuals with first‐ever, unilateral subcortical stroke into the experimental group (*n* = 26) and the control group (*n* = 26). Patients in the experimental group received 20 min of dual‐tDCS over the PSC and 40 min of sensorimotor training each session, while patients in the control group received sham dual‐tDCS. The treatment cycle was a 1‐h session of therapy each day, 5 days per week for 4 weeks. The Fugl–Meyer Assessment of Upper Extremity (FMA–UE) subscale, Action Research Arm Test (ARAT), Box and Block test (BBT), Erasmus MC revised Nottingham sensory assessment scale (Em‐NSA), Neurometer sensory nerve quantitative detector (CPT), the Barthel index (BI), and Hospital Anxiety and Depression Scale (HADS) were used to assess upper limb function, activities of daily living (ADL), and mental health before and after the 4‐week treatment period. In addition, functional near‐infrared spectroscopy (fNIRS) was used to explore potential clinical brain mechanisms.

**Results:**

Both groups showed significant improvement in all clinical scales (All *p* < 0.05) after treatment. Compared with sham‐tDCS plus sensorimotor training, active dual‐tDCS coupled with sensorimotor training can significantly improve the FMA‐UE, ARAT, Em‐NSA‐Stereognosis, and CPT‐2K Hz. In addition, dual‐tDCS combined with sensorimotor training can significantly activate the left pre‐Motor and supplementary motor cortex (PM–SMC) and enhance the functional connection between the left somatosensory association cortex (SAC) and RPM–SMC. Furthermore, the difference of FMA–UE in the experimental group was positively correlated with the functional connectivity of RPM‐SMC‐LSAC (*r* = 0.815, *p* < 0.001).

**Conclusion:**

Dual‐tDCS over the PSC combined with sensorimotor training can improve upper limb sensory and motor dysfunction, enhance ADL, and alleviate depression and anxiety for subacute stroke patients. Our results indicated that RPM‐SMC‐LSAC may be potential therapeutic targets for dual‐tDCS in upper limb rehabilitation on stroke.

## Graphical abstract

Dual‐tDCS combined with sensorimotor training can improve upper limb sensory and motor dysfunction, enhance ADL, alleviate depression and anxiety, and enhance the functional connectivity of sensorimotor functional brain cortex for subacute stroke patients.

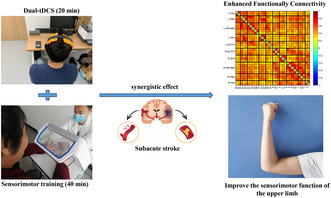

## 1 INTRODUCTION

Stroke is the third highest cause of mortality and is associated with the greatest disability‐adjusted life‐year in China.^1^ Post‐stroke survivors experience a variety of dysfunctions, with upper limb impairment being the most common.^2^ According to the observational study, up to 80% of patients will suffer from upper limb motor dysfunction after stroke,^3^ and about 50% of patients would have sensory dysfunction.^4^ Upper limb dysfunction after stroke is more difficult to recover than lower limb impairment, which will significantly affect patients' activities of daily life (ADL), reduce their quality of life, and increase the economic burden.^5^, ^6^ The first Stroke Recovery and Rehabilitation Roundtable (SRRR) established a new standard for stroke recovery research^7^: hyper‐acute phase (0–24 h), acute phase (1–7 days), subacute phase (7 days–6 months), and chronic phase (>6 months). Studies indicated that the subacute phase is a critical time for neural plasticity and should be a target for recovery trials.^8^, ^9^, ^10^ Therefore, how to help stroke patients recover upper limb sensorimotor function in the subacute stage is a hot issue in rehabilitation medicine at present.

Sensorimotor training, which is based on the principle of sensory integration, refers to promoting the recovery of sensorimotor function and forming new network connections through sensory stimulation and motor training with strong pertinence, appropriate and sufficient amount of intervention.^11^, ^12^ According to the degree and mode of patients' participation, sensorimotor training can be divided into passive sensorimotor training, active sensorimotor training, and mixed sensory‐motor training.^13^ The recently advanced studies indicated that skill‐based sensorimotor training and robot‐aided somatosensory training can improve somatosensory and motor function in stroke survivors.^14^, ^15^, ^16^, ^17^ In addition, our research group found that active sensorimotor training could significantly improve the upper extremity sensorimotor function of patients with subacute stroke compared with conventional rehabilitation training.^18^, ^19^

Transcranial direct current stimulation (tDCS) is a non‐invasive neuroregulatory technique that uses constant low‐intensity direct current (1–2 mA) to modulate neuronal activity in the cerebral cortex.^20^, ^21^ The excitability of the affected side decreased and the compensatory excitability of the unaffected side increased after unilateral stroke.^22^ Studies have shown that the anode tDCS can depolarize the neuronal potential of the brain to enhance cortical excitability, while the cathode can hyperpolarize the neuronal potential to reduce cortical excitability.^23^ Therefore, purposeful modulation of cortical excitability using tDCS may induce plastic changes within the network of sensorimotor areas of the cortex and improve the upper limb function of stroke patients. According to the position of the electrode, the stimulation mode of tDCS can be divided into unilateral‐anodal tDCS, unilateral‐cathode tDCS, and bilateral dual‐tDCS.^24^ Studies have shown that bilateral dual‐tDCS can more effectively regulate cortical excitability and improve upper limb function than unilateral tDCS.^25^, ^26^, ^27^ Furthermore, a recent systematic review and meta‐analysis indicated that stimulation of the primary somatosensory cortex (PSC) showed significant therapeutic potential to improve sensorimotor function in patients with stroke.^28^

Accordingly, dual‐tDCS over PSC has the potential to amend inter‐hemispheric imbalance in subacute stroke. We hypothesized that dual‐tDCS over PSC could enhance the benefits of sensorimotor training, by favoring the restoration of interhemispheric balance in subacute stroke patients. To investigate this question, a double‐blind randomized controlled trial that compared combined dual‐tDCS and sensorimotor training with sham‐tDCS plus sensorimotor training was performed. The objective of this article is twofold: first, to study the effectiveness of dual‐tDCS over PSC combined with sensorimotor training on subacute stroke patients; and second, to explore the potential clinical brain mechanism of this combination therapy by using the functional near‐infrared spectroscopy neuroimaging (fNIRS).

## 2 MATERIALS AND METHODS

### 2.1 Study design

In this randomized, double‐blinded, sham‐controlled study, subacute stroke patients received either 20 sessions of dual‐tDCS, or sham‐tDCS combined with sensorimotor training. The allocation ratio was 1:1. This study was approved by the Institutional Review Board of Huashan Hospital, Fudan University (No. KY2021‐815) and registered at the Chinese Clinical Trial Registry (ChiCTR2100052663).

### 2.2 Participants

The subacute stroke patients included in this study were consecutively recruited from Huashan Hospital, Fudan University between August 2021 and November 2022. The inclusion criteria were the following: first‐ever stroke confirmed by CT or MRI, chronicity ≥14 and <180 days, Mini‐Mental State Examination (MMSE)^29^ score ≥ 20, and aged between 18 and 70 years. Patients were excluded if they presented one of the following: history of seizures, presence of neural implants, use of a pacemaker, moderate‐to‐severe spasm of extensor and flexor muscles of hand and wrist (modified Ashworth^30^ score ≥2), aphasia or serious dysarthria.

### 2.3 Sample Size

We use PASS software (NCSS Statistical Software) to calculate the required sample size. The Fugl–Meyer Assessment for Upper Extremity (FMA–UE)^31^ subscale was considered significant when the change value was more than 5 points. A power analysis indicated the necessary sample size to be *n* = 20 per group with an *α* level of 0.05 and a power of 0.95. Considering a dropout rate of 20%, we aimed to include 25 patients in each of the two groups.

### 2.4 Randomization

Participants were stratified according to the FMA–UE score and were randomly divided into the experimental group or the control group by using a computer‐generated random number of sequences. An independent researcher not involved in the study created the blocked randomization sequence.

### 2.5 Interventions

All the participants were invited for an initial assessment to confirm that they met the inclusion criteria. After giving informed consent, eligible participants were allocated to 1 of 2 groups. Both groups underwent 20 sessions of dual‐tDCS and sensorimotor training or sham‐tDCS and sensorimotor training for 1 h, 5 times a week for 4 weeks (excluding weekends). Studies have shown that tDCS has an obvious after effect and the increase of MEP after tDCS intervention can still be detected 60 min after stimulation.^32^, ^33^ Therefore, in this study, patients were given 20 min tDCS and then received 40 min sensorimotor training.

#### 2.5.1 Transcranial direct current stimulation

ActivaDose®II portable transcranial direct current stimulator (ActivaTek Company, the United States) was used to intervene in the central nervous system of subacute stroke patients. The motor‐evoked potentials module of transcranial magnetic stimulation (TMS) equipment (Model: YRD‐CCY1; Wuhan Yiruide Medical Equipment Co., Ltd., Wuhan, China, YZB‐20142211249) is used to locate M1, and the best stimulation point of M1 is found by adjusting the intensity and direction of the stimulation coil. The rear 2 cm parallel to the midline of M1 is the best stimulation point of the PSC.

In this study, the bilateral stimulation mode of tDCS was used. The cathode was positioned over the contralesional PSC, while the anode was placed over the ipsilesional PSC.^34^ The electrode piece was a 5 cm × 5 cm isotonic saline sponge electrode, the total current intensity was 2 mA, and the treatment time was 20 min/time. For tDCS sham stimulation, we only turn on the instrument in the first 30 s and turn it off later.

#### 2.5.2 Sensorimotor training

**FIGURE 1:**
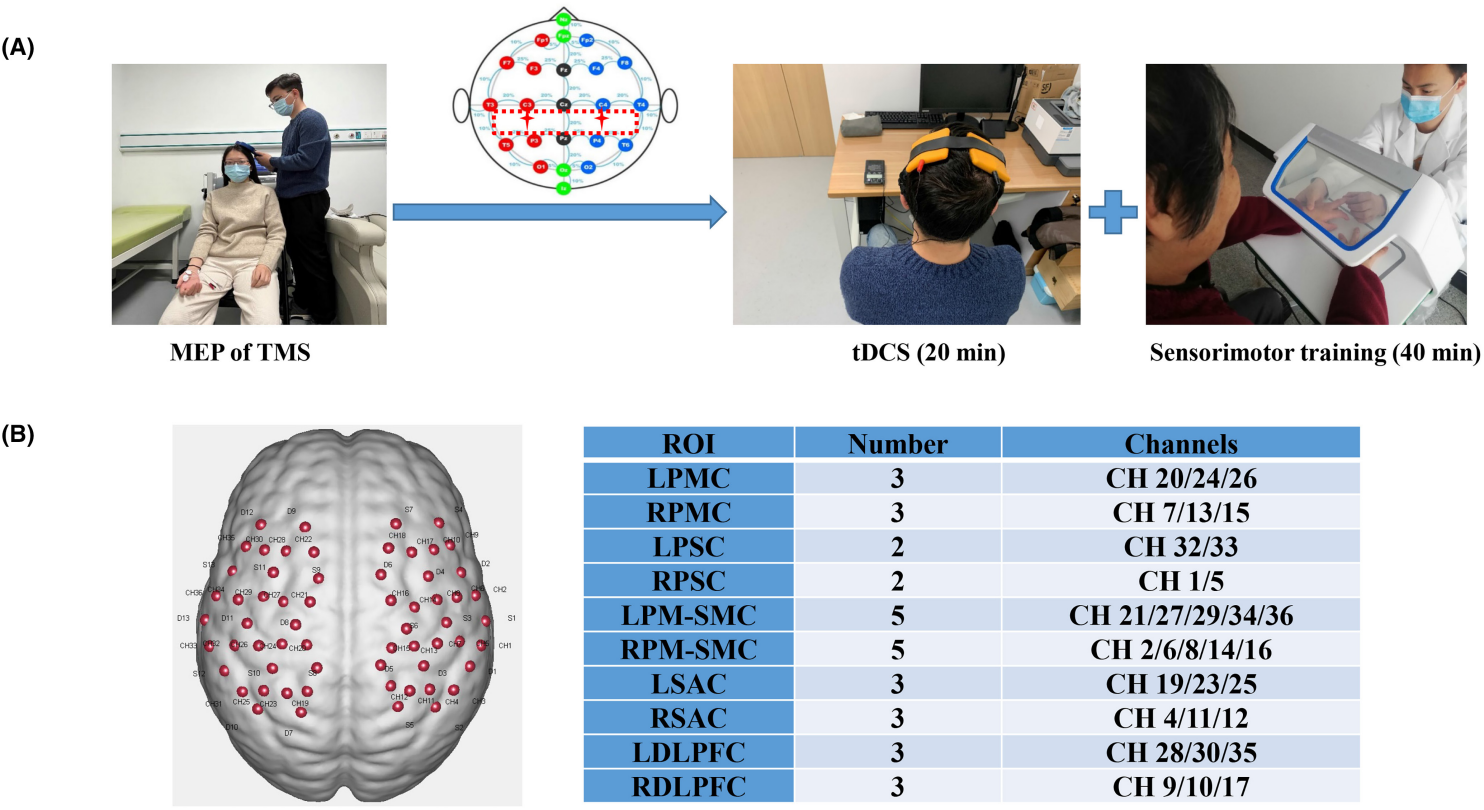
Experimental procedure and distribution map of fNIRS channels. A: The target brain area (primary somatosensory cortex) was located by the MEP module of the TMS instrument, and then 20 min of tDCS stimulation and 40 min of sensorimotor training were performed. B: fNIRS 36 channels distribution map and regions of interest map. PMC, primary motor cortex; PSC, primary somatosensory cortex; PM–SMC, pre‐Motor and supplementary motor cortex; SAC, somatosensory association cortex; DLPFC, the dorsolateral prefrontal cortex.

The hand–brain perception device (Model: SensiTouch2; Shanghai Electric Intelligent Rehabilitation Medical Co., Ltd., Shanghai, China) was used to train the upper limb sensorimotor function for included patients. The device can be used for visual masking, which can more effectively help patients restore the sensorimotor function of the upper limb.^18^ This study uses a three‐step method to train patients' sensorimotor functions: the first step is to conduct sensorimotor assessment; the second step is to perform passive sensory stimulation and active sensory training; the third step is to perform task‐oriented motor function training for patients. Sensorimotor training was tailored to meet all patients' deficits and lasted a total of 40 min per day (Figure 1A).

### 2.6 Outcome measures

The outcome indexes of this study were mainly evaluated from the aspects of upper limb motor function, sensory function, ADL, mental health, and functional near‐infrared brain function. All the evaluation was completed within 1 day, with a total time of 2 h. Outcomes were measured before and after 4 weeks of treatment.

#### 2.6.1 Motor function assessment

Two effective clinical‐based scales and one test were selected to evaluate the upper limb motor function of included patients. The primary outcome was the FMA–UE. FMA–UE is the most commonly used scale for evaluating upper limb motor function in patients with stroke, and it has good reliability and validity. FMA–UE consists of 33 items, with a total score of 66. The higher the score, the closer to the normal function of the patient.^35^, ^36^, ^37^ In addition, the Action Research Arm Test (ARAT) and Box and Block test (BBT) were selected as second motor outcomes. The ARAT evaluated the fine motor function of the upper limbs from four basic movements: grasp, grip, pinch and gross movement, including 19 items with a total score of 57. The higher the score, the better the fine motor function of the upper limb.^38^ The BBT was used to assess the gross motor function of the upper limbs and hands. The evaluation method was as follows: The patient was required to move the building blocks from one side of the box to the other side of the box as quickly and as many times as possible within 60 s. The average of the patient's three times was taken as the patient's score.^39^

#### 2.6.2 Somatosensory function assessment

The Erasmus‐MC revised Nottingham sensory assessment scale (Em–NSA) and quantitative sensory nerve detector were used to assess the change in sensory function for patients. Em–NSA can be used to evaluate patients' tactile, proprioceptive, and stereognosis perception with good reliability and validity.^40^ Neurometer sensory nerve quantitative detector (CPT) was used to evaluate the sensory fibers' sensitivity of the patients. The CPT generates a constant current stimulus that evokes responses that quantify the functional integrity of each of the three major sub‐populations of sensory nerve fibers. Specifically, Aβ, Aδ, and C fiber groups are selectively stimulated by sinusoid waveform currents of 2 K, 250, and 5 Hz, respectively.^41^ In this study, the device was used to evaluate the sensory threshold of the affected index finger.

#### 2.6.3 ADL and HADS assessment

The Barthel index (BI) is used to evaluate patients' ADL levels, which includes 10 items with a total score of 100. A higher score indicated a greater ability to function independently.^42^ Furthermore, the Hospital Anxiety and Depression Scale (HADS) was used to assess the degree of anxiety and depression in patients, which consists of 7 anxiety scoring items and 7 depression scoring items. The higher the score, the more severe the anxiety and depression of the patient.^43^

#### 2.6.4 Functional near‐infrared spectroscopy neuroimaging

We used an fNIRS system (NirScan, Danyang Huichuang Medical Equipment Co. Ltd., China) with wavelengths of 695 and 830 nm. The sampling frequency was set to 20 Hz. Based on the international EEG 10/20 system, The fNIRS cap setup included 13 emitters of near‐infrared light and 13 detectors spaced 3 cm apart, yielding 36 data channels deployed at the sensorimotor brain area. According to the coordinate information, some of the 36 channels were classified into 5 regions of interest (ROI) in the left and right brain: primary motor cortex (PMC), PSC, pre‐Motor and supplementary motor cortex (PM–SMC), somatosensory association cortex (SAC), and dorsolateral prefrontal cortex (DLPFC) (Figure 1B).

Before the fNIRS test began, the patients were allowed to rest for 5 min in a quiet environment to relax. After resting, the patients took a comfortable sitting position and began the test task after completing the data calibration. For the resting state, they were relaxed with their eyes closed for 5 min in a quiet room. The participants were reminded to stay awake and then perform the motor task. In the quiet room, the patients sat with their hands on their legs naturally, and according to the instructions of the system, they opened and clenched their hands at the same time, repeated for 15 s, and rested for the 20 s, a total of 3 groups.

### 2.7 Analysis of fNIRS data

Matlab2014a software (The Mathworks, USA) was used to analyze fNIRS data. Firstly, the patient's original data file was converted into a format, and then the data were preprocessed. The general linear model (GLM) is used to model the data, estimate the parameters, and get the *β* value, based on which the interested experimental effects are extracted and the contrast vector is set. After the experimental effect of ROI was obtained by using the above method on each test, it can be involved in the group‐level statistical analysis. The hemodynamic response induced by experimental conditions of fNIRS data was predicted, and then the activation of cerebral blood flow induced by each experimental condition was calculated according to the estimated results of model parameters. The resting state data analysis was based on seed point correlation analysis, whole brain correlation analysis, or independent component analysis to evaluate brain functional connectivity.

### 2.8 Statistical analysis

Statistical analysis was performed using SPSS 25.0 (IBM Corporation, Armonk, New York, NY, USA). Data were confirmed to have a normal distribution using the Shapiro–Wilk test. Data are presented as the mean ± standard deviation (SD) for normally distributed continuous variables. Nonnormally distributed variables or ranked variables are expressed as medians (interquartile ranges, IQRs). Two‐tailed *t*‐tests (for continuous variables) and the chi‐square test (for categorical variables) were used to compare baseline measures between the 2 treatment groups. Nonparametric tests were used if the data seemed to be nonnormally distributed. The Wilcoxon signed‐rank test was used for within‐group analyses, and the Mann–Whitney test was used for between‐group analyses. We correlated any significant changes between brain activity and behavioral clinical scales by using Pearson correlation analysis. The level of significance was set at 0.05.

## 3 RESULTS

### 3.1 Participant characteristics

**FIGURE 2:**
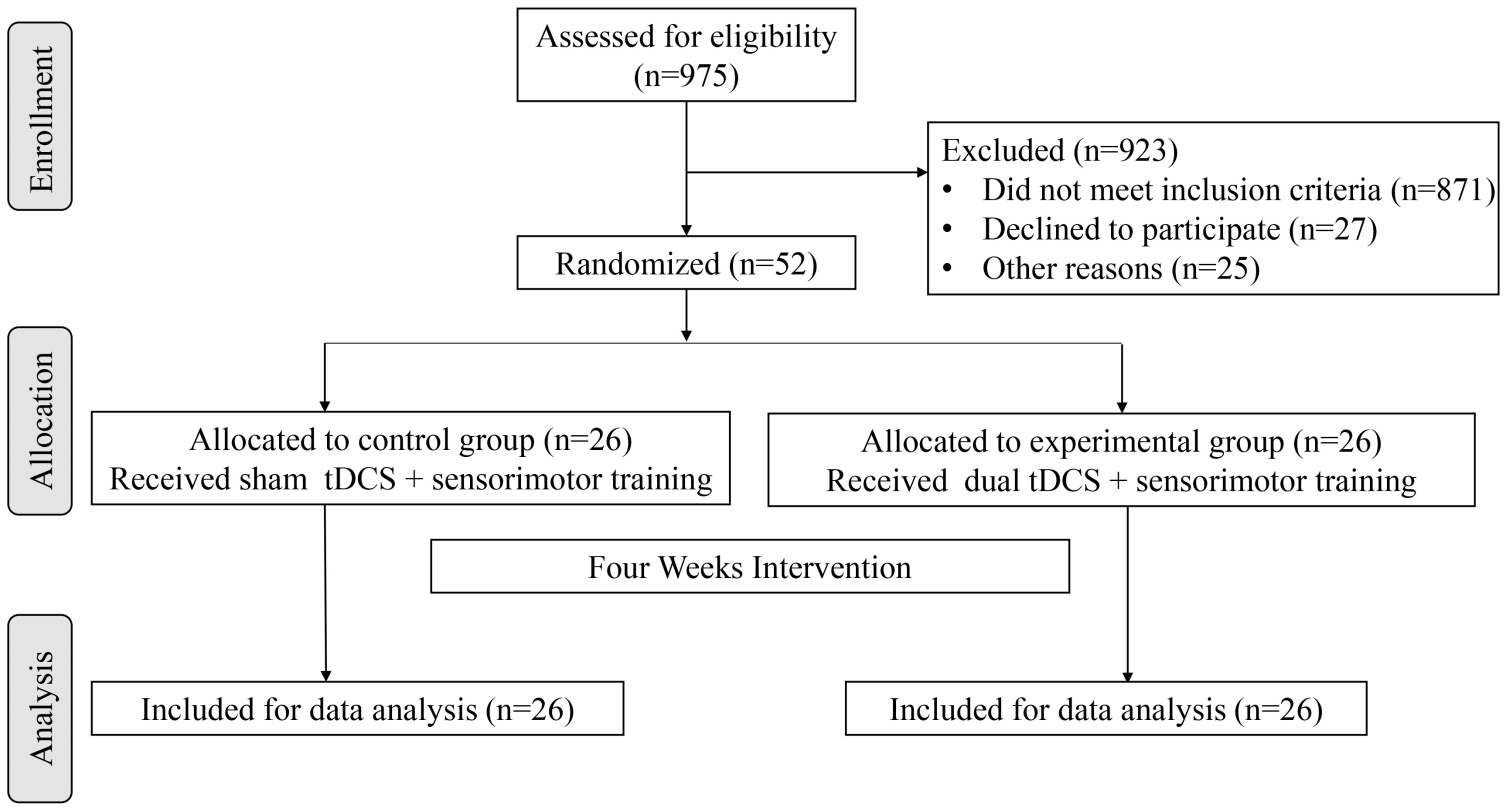
Flow chart for participant selection and assignment.

The flowchart for participants is presented in Figure 2. A total of 975 hospitalized patients in Huashan Hospital were assessed for eligibility and we finally included 52 patients. The participants' baseline demographic characteristics are shown in Table 1. No significant differences were observed between the groups regarding age, gender, stroke type, affected side, and time from onset. In addition, there were no significant differences between the two groups in clinical outcomes and brain function at baseline.

**TABLE 1:** Baseline demographic and clinical characteristics of the patients.

Variable	EG (*N* = 26)	CG (*N* = 26)	*t* or *z* value	*p* Value
Age, years	58.65 ± 12.677	56.54 ± 10.428	0.657^a^	0.514
Gender, *n* (%)			0.495^b^	0.482
Male	22 (84.6)	20 (76.9)		
Female	4 (15.4)	6 (23.1)		
Stroke type, *n* (%)			0.000^b^	1.000
Ischemic	18 (69.2)	18 (69.2)		
Hemorrhagic	8 (30.8)	8 (30.8)		
Affected side, *n* (%)			0.702^b^	0.402
Left	13 (50)	10 (38.5)		
Right	13 (50)	16 (61.5)		
Time from the onset, days	100.12 (56.25–151.75)	87.58 (61.75–98.75)	−0.623^c^	0.533
MMSE	25.92 (24.00–28.00)	25.27 (24.75–27.00)	−0.660^a^	0.509
Brunnstrom‐U	2.92 (1.00–5.00)	2.92 (1.00–5.00)	0.000^a^	1.000
FMA–UE	25.81 (8.00–43.25)	25.42 (5.50–48.00)	−0.257^a^	0.797
ARAT	14.12 (0.00–26.00)	12.77 (0.00–36.25)	−0.371^a^	0.711
BBT	5.12 (0.00–9.25)	7.12 (0.00–14.25)	−0.193^a^	0.847
Em‐NSA‐Light touch	60.58 (51–76)	58.46 (60–80.25)	1.306^a^	0.192
Em‐NSA‐Proprioception	6.08 (4.00–8.00)	7.00 (4.00–8.00)	1.670^a^	0.095
Em‐NSA‐Stereognosis	5.46 (4.00–7.00)	6.54 (4.00–9.00)	1.803^a^	0.071
CPT‐2000 Hz	333.10 (207.50–482.75)	398.53 (227.00–462.00)	0.731^a^	0.478
CPT‐250 Hz	150.20 (99.50–202.50)	176.21 (91.00–182.00)	−0.604^a^	0.550
CPT‐5 Hz	119.35 (65.25–156.75)	159.00 (52.00–197.00)	−0.351^a^	0.728
BI	60.58 (53.75–70.00)	58.46 (45.00–71.25)	−0.630^a^	0.529
HADS	10.42 (6.75–14.00)	9.04 (3.00–14.00)	−1.128^a^	0.259

Abbreviations: ARAT, Action Research Arm Test; BBT, Box and Block test; BI, Barthel index; Brunnstrom‐U, Brunnstrom stage‐upper extremity; CG, Control group; CPT, current perception threshold; EG, Experimental group; Em‐NSA, Erasmus MC revised Nottingham sensory assessment scale; FMA–UE, Fugl–Meyer Assessment of the Upper Extremity; HADS, Hospital Anxiety and Depression Scale; MMSE, Mini‐Mental State Examination.

^a^ Two‐tailed *t*‐test.

^b^ Chi‐square test.

^c^ Wilcoxon rank sum test.

### 3.2 Clinical outcomes

#### 3.2.1 Effect on the motor function

**FIGURE 3:**
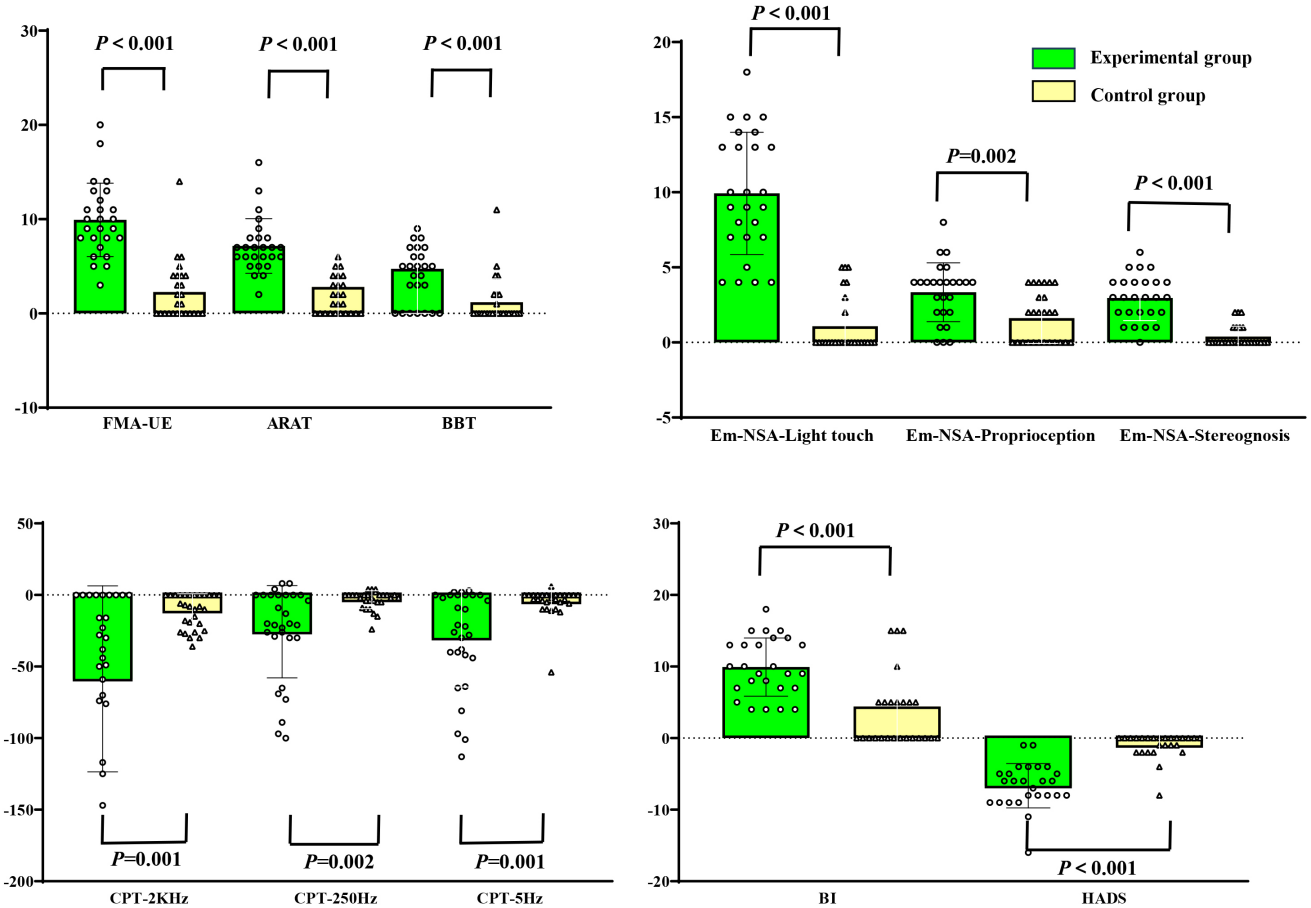
Comparison of Longitudinal changes in clinical outcomes between the experimental group and the control group. A: FMA–UE, Fugl–Meyer Assessment of the Upper Extremity; ARAT, Action Research Arm Test; BBT, Box and Block test. B: Em‐NSA, Erasmus MC revised Nottingham sensory assessment scale. C: CPT, current perception threshold. D: BI, Barthel index; HADS, Hospital Anxiety and Depression Scale.

The main result of our study was that both groups showed significant improvement in the FMA‐UE, ARAT, and BBT scores over time. The ARAT score of the experimental group after treatment was significantly higher than that of the control group (*p* = 0.039) (Table 2). In addition, the experimental group's mean score of the FMA–UE, ARAT, and BBT increased by 9.92, 7.15, and 4.73, respectively, after the intervention, which was significantly higher than the corresponding increase in the control group (Figure 3A).

**TABLE 2:** Comparison of clinical outcomes in the experimental group and control group.

Variable/Group	Pre‐intervention	Post‐intervention	*t* or *z* value	*p‐*Value
FMA–UE			−1.585	0.113
EG	25.81 (8.00–43.25)	36.92 (16.50–56.00)	−4.377	<0.001
CG	25.42 (5.50–48.00)	27.69 (7.00–51.25)	−3.307	0.001
ARAT			−2.065	0.039
EG	14.12 (0.00–26.00)	21.96 (6.00–36.00)	−4.388	<0.001
CG	12.77 (0.00–36.25)	15.58 (0.00–40.00)	−3.526	<0.001
BBT			−1.378	0.168
EG	5.12 (0.00–9.25)	10.24 (1.50–16.00)	−3.835	<0.001
CG	7.12 (0.00–14.25)	8.31 (0.00–19.25)	−2.536	0.011
Em‐NSA‐Light touch			−0.752	0.452
EG	60.58 (51–76)	73.16 (63.50–84.50)	−4.379	<0.001
CG	58.46 (60.00–80.25)	69.42 (60.00–82.00)	−2.388	0.017
Em‐NSA‐Proprioception			−0.932	0.351
EG	6.08 (4.00–8.00)	9.48 (8.00–12.00)	−4.155	<0.001
CG	7.00 (4.00–8.00)	8.62 (6.75–12.00)	−3.355	0.001
Em‐NSA‐Stereognosis			−2.337	0.019
EG	5.46 (4.00–7.00)	8.52 (8.00–9.00)	−4.307	<0.001
CG	6.54 (4.00–9.00)	6.92 (4.00–9.00)	−2.428	0.015
CPT‐2K Hz			−2.333	0.020
EG	333.10 (207.50–482.75)	241.84 (167.00–322.00)	−3.824	<0.001
CG	398.53 (227.00–462.00)	382.05 (204.00–455.00)	−3.518	<0.001
CPT‐250 Hz			−0.562	0.574
EG	150.20 (99.50–202.50)	113.68 (78.00–121.00)	−3.744	<0.001
CG	176.21 (91.00–182.00)	170.58 (87.00–178.00)	−3.192	0.001
CPT‐5 Hz			−0.971	0.332
EG	119.35 (65.25–156.75)	75.95 (41.00–95.00)	−3.823	<0.001
CG	6.54 (4.00–9.00)	6.92 (4.00–9.00)	−2.428	0.015
BI			−2.452	0.014
EG	60.58 (53.75–70.00)	73.60 (65.00–87.50)	−4.322	<0.001
CG	58.46 (45.00–71.25)	62.88 (48.75–80.00)	−3.133	0.002
HADS			−2.101	0.036
EG	10.42 (6.75–14.00)	3.56 (1.00–5.00)	−4.383	<0.001
CG	9.04 (3.00–14.00)	8.04 (1.75–14.00)	−2.979	0.003

Abbreviations: ARAT, Action Research Arm Test; BBT, Box and Block test; BI, Barthel index; CPT, current perception threshold; Em‐NSA, Erasmus MC revised Nottingham sensory assessment scale; FMA–UE, Fugl–Meyer Assessment of the Upper Extremity; HADS, Hospital Anxiety and Depression Scale.

#### 3.2.2 Effect on the sensory function

Significant within‐patient improvements were noted in both groups for the Em‐NSA and CPT scores between pre‐assessment and post‐assessment. The Em‐NSA‐Stereognosis and CPT‐2K Hz scores of the experimental group after treatment were significantly higher than that of the control group (Em‐NSA‐Stereognosis: *p* = 0.019; CPT‐2K Hz: *p* = 0.02; Table 2). In addition, the difference between EM‐NSA‐Stereognosis and CPT‐2KHz before and after treatment showed that the improvement of the experimental group was significantly better than that of the control group (*p <* 0.05; Figure 3B,C).

#### 3.2.3 Effect on the ADL and mental health

Significant differences between pre‐intervention and post‐intervention assessments were observed for the BI and HADS in both the groups (Table 2). Furthermore, the improvement degree of BI and HADS scores after treatment and the difference before and after treatment in the experimental group was significantly better than that in the control group (*p <* 0.05; Figure 3D).

#### 3.2.4 Brain activation and functional connectivity changes

The comparison in the experimental group showed that channel 34 (*T* = 2.46, *p* = 0.028) and channel 36 (*T* = 2.19, *p* = 0.045) were significantly activated after treatment. Analysis of resting‐state data showed that functional connectivity between LSAC and RPM–SMC was significantly enhanced (*T* = 2.06, *p* = 0.048; see Figure S1).

**FIGURE 4:**
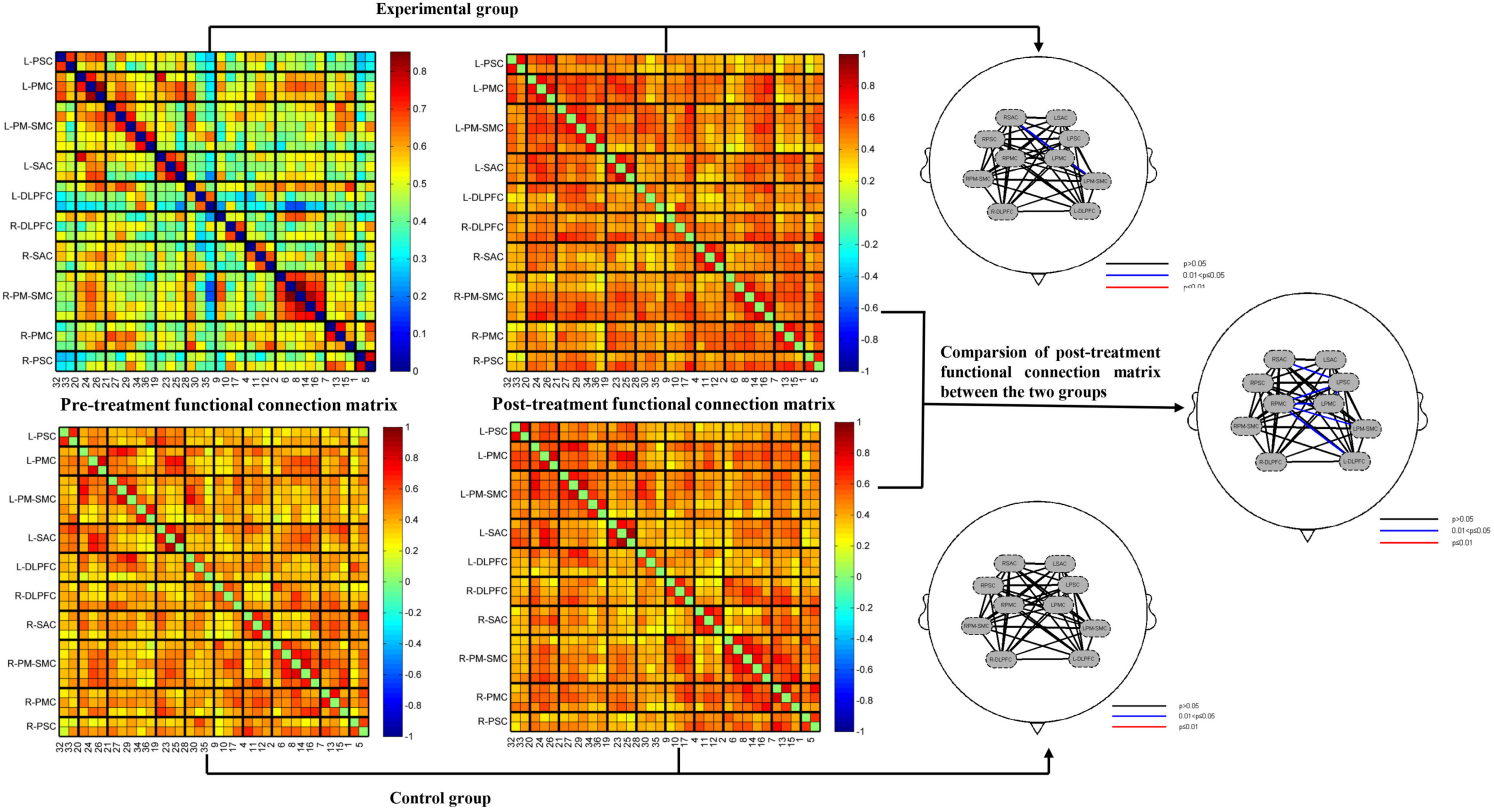
The functional connection matrix of the experimental group and the control group before and after treatment.

Analysis of fNIRS motor task data in the control group showed that channel 18 was significantly activated after treatment (*T* = 2.30, *p* = 0.040) (see Figure S2). However, the functional connection between the ROI in the control group was not statistically significant (Figure 4).

The comparison between the two groups showed that the experimental group was significantly activated in channel 10 (*T* = 2.07, *p* = 0.047), channel 12 (*T* = 2.59, *p* = 0.014), channel 15 (*T* = 2.36, *p* = 0.024), and channel 18 (*T* = 2.25, *p* = 0.031) compared with the control group after treatment. In addition, resting‐state data analysis showed that the experimental group had enhanced functional connectivity within and between ROIs compared to the control group after treatment. There were significant differences in functional connectivity within some ROIs: RPSC–RPSC (*T* = 2.67, *p* = 0.016), RPMC–RPMC (*T* = 2.31, *p* = 0.031), LPMC–LPMC (*T* = 2.09, *p* = 0.046), and RDLPFC–RDLPFC (*T* = 2.45, *p* = 0.024). Significant differences were observed in connectivity between some ROIs: LPSC–RPMC (*T* = 2.34, *p* = 0.026), LPSC–LPMC (*T* = 2.09, *p* = 0.048), LPSC–RSAC (*T* = 2.14, *p* = 0.041), RPMC–LPMC (*T* = 2.63, *p* = 0.013), RPMC‐LPM‐SMC (T = 2.35, *p* = 0.028), and RPMC–LDLPFC (*T* = 2.21, *p* = 0.040; Figure 4).

#### 3.2.5 Brain‐behavior correlations

**FIGURE 5:**
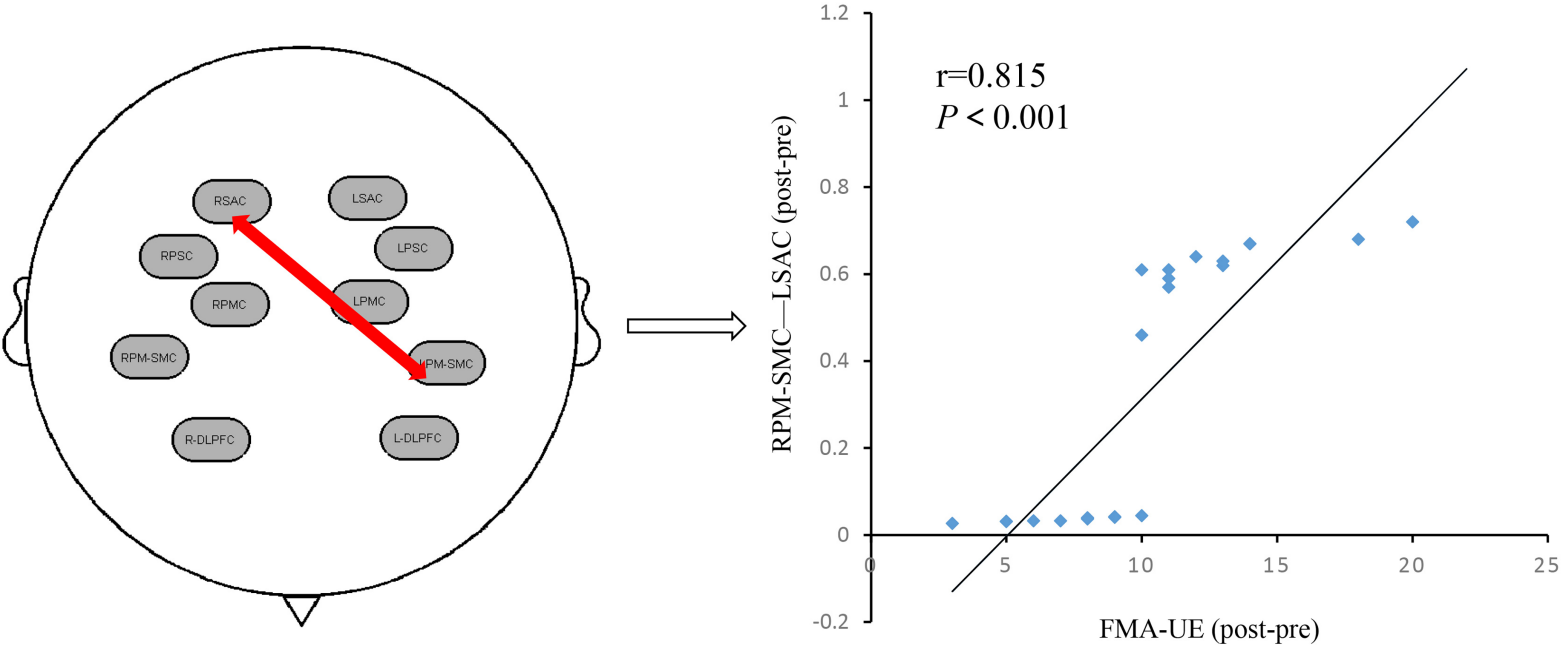
Correlation analysis between the difference of FMA–UE and the difference of functional connection strength of LPM–SMC and RSAC before and after treatment in the experimental group.

By correlation analysis of clinical scales and brain function data of fNIRS, we found that the difference of FMA–UE in the experimental group was positively correlated with the functional connectivity of RPM‐SMC‐LSAC (*r* = 0.815, *p* < 0.001; Figure 5). However, there was no significant correlation between the functional connections between other ROIs and the scale evaluation.

### 3.3 Safety

No significant adverse events occurred during the clinical research. Reported adverse events were four participants in the active dual‐tDCS reported mild tingling that persisted for the first stimulation, which was foreseen and already mentioned to the patients before therapy began.

## 4 DISCUSSION

This double‐blind randomized controlled study investigated if the addition of dual‐tDCS over the PSC to sensorimotor training would promote greater positive on sensorimotor function compared with sham tDCS in subacute stroke patients. Our results showed that dual‐tDCS combined with sensorimotor training significantly improved sensory function, motor performance, and ADL compared with sham‐tDCS plus sensorimotor training. In addition, the results indicated that dula‐tDCS plus sensorimotor training could release the degree of depression and anxiety for subacute stroke patients. Furthermore, fNIRS results proved that dual‐tDCS in combination with sensorimotor training can significantly activate the LPM–SMC and enhance the functional connectivity between LSMC and RPM–SMC for subacute stroke patients.

The synergistic effect of dual‐tDCS coupled with sensorimotor training found in our study is in line with previously published articles. Lefebvre et al. found that a single session of dual‐tDCS combined with motor skill learning can significantly enhance online skill learning for chronic stroke patients.^44^, ^45^ The results of Dehem et al.^46^ showed a slight improvement in hand dexterity and arm movement after the dual‐tDCS plus robotic‐assisted therapy. Takebayashi et al.^47^ presented that dual‐tDCS combined with constraint‐induced movement therapy can significantly improve FMA–UE scores in patients with chronic stroke. Hsu et al.^26^ indicated that bihemispheric tDCS during task‐oriented training may facilitate motor recovery in subacute stroke patients. In addition, our research group's previous research also demonstrated that dual‐tDCS combined with task‐oriented training can more effectively improve upper limb FMA–UE scores in chronic phase patients compared to sham stimulation.^48^ The results of this study indicate that dual‐tDCS combined with sensorimotor training can significantly improve the ARAT score of patients compared to the sham stimulation group, which is consistent with the above research. These results suggest that dual‐tDCS has a synergistic effect on peripheral rehabilitation training.

Previous studies on dual‐tDCS in stroke mainly focused on the motor function recovery of patients, while studies on sensory function rehabilitation were still lacking. Fujimoto et al.^34^ concluded that dual‐hemisphere tDCS over the primary and secondary somatosensory cortices can transiently enhance tactile discriminative task performance in chronic stroke patients with sensory dysfunction. A recent study published in *Brain Stimulation* indicated that dual‐tDCS can significantly induce the reaching and coordination tasks while proprioception compared with anodal‐tDCS.^25^ The results of our study showed that dual‐tDCS on the primary somatosensory cortex combined with sensorimotor training could significantly improve patients' light touch, proprioception, and stereognosis. In addition, previous studies mainly used semi‐quantitative clinical scales to evaluate the somatosensory function of patients. To improve the shortcomings of the above studies, a neurometer sensory nerve quantitative detector was used in this study to quantitatively assess the somatosensory function of patients. The results of this study showed that dual‐tDCS coupled with sensorimotor training can significantly enhance the sensitivity of Aβ fibers (CPT 2KHZ) compared with the sham group. Previous observational studies suggested that the sensitivity of Aβ fibers was significantly decreased after bilateral carotid artery occlusion^49^ and middle cerebral artery occlusion.^50^ Our results showed that dual‐tDCS combined with sensorimotor training can improve the somatosensory function of subacute stroke patients by improving fiber sensitivity.

The results of this study indicate that dual‐tDCS combined with sensorimotor training can not only improve the upper limb sensorimotor function of subacute stroke patients but also relieve anxiety and depression symptoms of patients. A recent systematic review and meta‐analysis indicated that tDCS has an effect on improvement in post‐stroke depression.^51^ Our results are in line with this conclusion. There are two possible explanations for these clinical effects. On the one side, the relief of anxiety and depression in patients may be due to the improvement of their function. On the other side, this study found that dual‐tDCS combined with sensorimotor training can activate the DLPFC region of the brain, which is mainly related to the mental function of patients. Therefore, activation of the DLPFC may be the potential brain mechanism for relieving anxiety and depression in subacute stroke patients. However, one RCT study showed no add‐on effect of tDCS on depression for chronic stroke survivors.^52^ The results of our study are not consistent with those of this study, which suggests that tDCS in the subacute phase may be more effective in relieving depressive symptoms in stroke patients than in the chronic phase.

Previous studies have used TMS, MEG, and fMRI to preliminarily explore the clinical neural mechanisms of dual‐tDCS in the treatment of stroke. Kuo et al.^27^ indicated that task‐concurrent dual‐tDCS in subacute stroke can safely and effectively modulate bilateral M1 excitability and inter‐hemispheric imbalance and also movement‐related α‐activity. In addition, Lee et al.^53^ suggested that dual‐tDCS significantly increased interhemispheric connectivity between bilateral hemispheres, global efficiency of the motor network, and the interhemispheric connectivity of the contralesional M1 in subacute stroke patients. These studies suggest that dual‐tDCS can regulate cortical excitability and help patients reorganize their brain networks. Compared with EEG and fMRI, fNIRS has both good temporal and spatial resolution and is easier to carry and operate. Therefore, this study used fNIRS to explore the potential neural mechanism of dual‐tDNS combined with sensorimotor training. The results of this study presented that dual‐tDCS combined with sensorimotor training can significantly activate the LPM–SMC and enhance the functional connection between LSMC and RPM–SMC of subacute stroke patients. Our results suggest that bilateral tDCS combined with sensorimotor training can enhance the patient's sensorimotor network and promote the reorganization of the patient's brain network. In addition, this study also found that the connectivity strength of LPM‐SMC‐RSAC was positively correlated with the difference of FMA–UE. The result indicates that LPM and SMC–RSAC may be potential therapeutic targets in the future.

### 4.1 Clinical implications and future directions

Current findings suggest that dual‐tDCS over PSC combined with sensorimotor training is a promising approach for the improvement of upper limb sensory and motor function in subacute stroke patients. In addition, the results of this study indicate that RPM‐SMC‐LSAC may be potential therapeutic targets for clinical upper limb functional rehabilitation, and related research can be carried out in the future. Furthermore, the therapeutic effects of dual tDCS on different types of stroke can be explored. In addition, follow‐up and evaluation of patients are needed to explore the subsequent efficacy of this combination therapy.

### 4.2 Limitations

There are a few limitations in this study. The type and severity of stroke may affect clinical efficacy, but this study did not make a more detailed distinction. In addition, due to the limitation of research conditions, this study is the absence of a neuronavigation system that can target the primary somatosensory cortex according to the functional imaging examination.

## 5 CONCLUSION

In conclusion, dual‐tDCS over the PSC combined with sensorimotor training can improve upper limb sensory and motor dysfunction, enhance ADL, and alleviate depression and anxiety for subacute stroke patients. In addition, dual‐tDCS coupled with sensorimotor training can significantly activate the LPM–SMC and enhance the functional connection between LSMC and RPM–SMC. Current findings encourage the combination of dual‐tDCS with sensorimotor training for the development of enhanced upper limb function intervention in subacute stroke.

## AUTHOR CONTRIBUTIONS

Chong Li and Yun Chen: Conceptualization; methodology; investigation; writing—original draft. Shuting Tu, Yifang Lin, and Shuo Xu: Methodology; investigation. Jiaying Lin, Maohou Wu, and Yong Xie: Methodology. Jie Jia: Conceptualization; supervision; writing—review and editing.

## FUNDING INFORMATION

The study was supported by Joint Funds for the innovation of science and Technology, Fujian province (No. 2021Y9130), the Key National Research and Development Program (No. 2018YFC2002300 and 2018YFC2002301), the National Nature Innovation Research Group Project (No. 82021002), and the National Nature Integration Project (No. 91948302).

## CONFLICT OF INTEREST STATEMENT

The authors declare no competing financial interests.

## Supporting information


Figure S1

Figure S2

## Data Availability

The data that support the findings of this study are available from the corresponding author upon reasonable request.
