# Supplementary figures and images for: Dual‐tDCS combined with sensorimotor training promotes upper limb function in subacute stroke patients: A randomized, double‐blinded, sham‐controlled study
Source: CNS Neurosci Ther. 2023 Nov 23;30(4):e14530. doi: 10.1111/cns.14530 (PMC11017427; doi:10.1111/cns.14530)

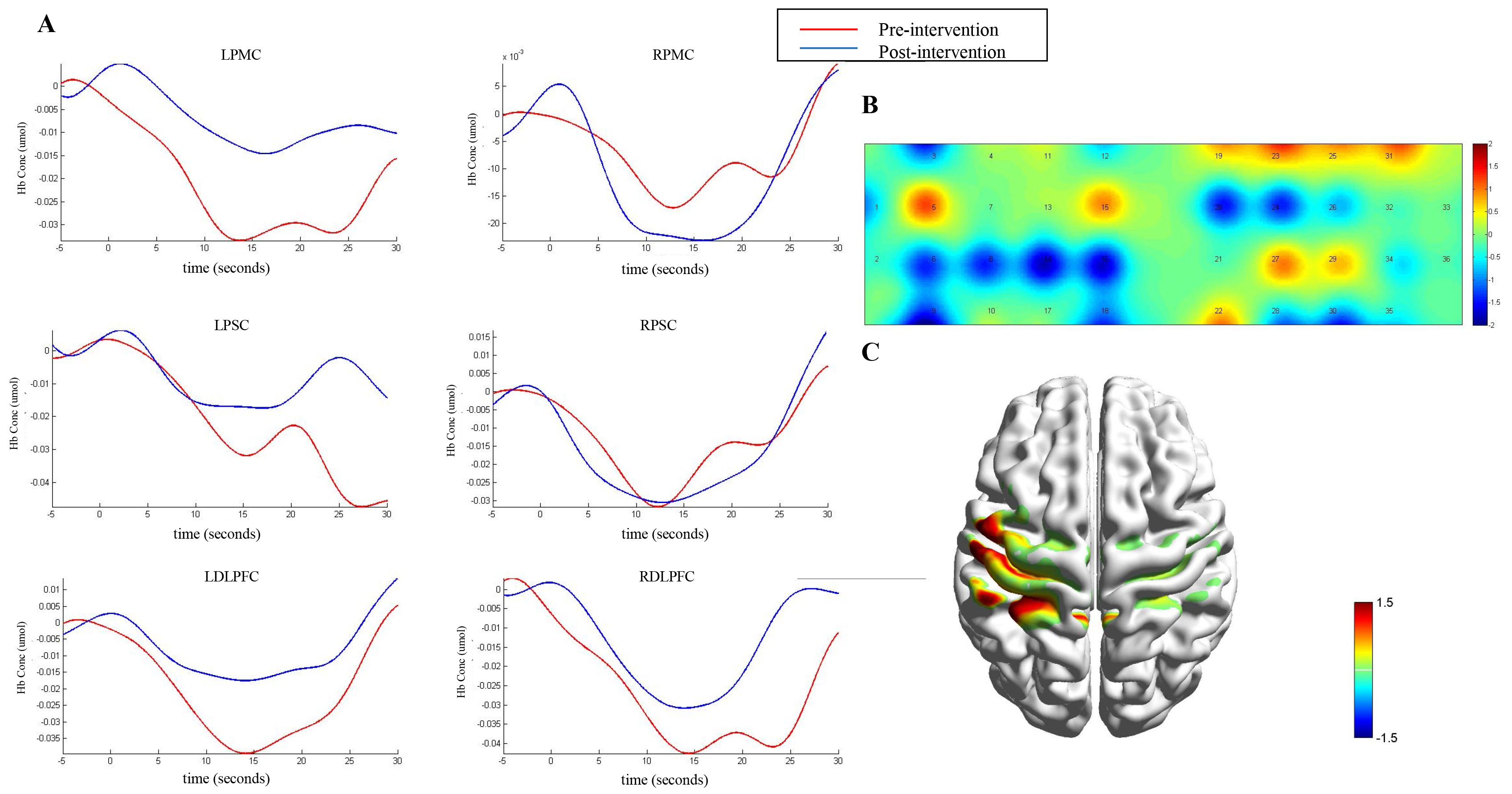

Supplement: Supplementary file 1 — Figure S1 [file CNS-30-e14530-s002.jpg]

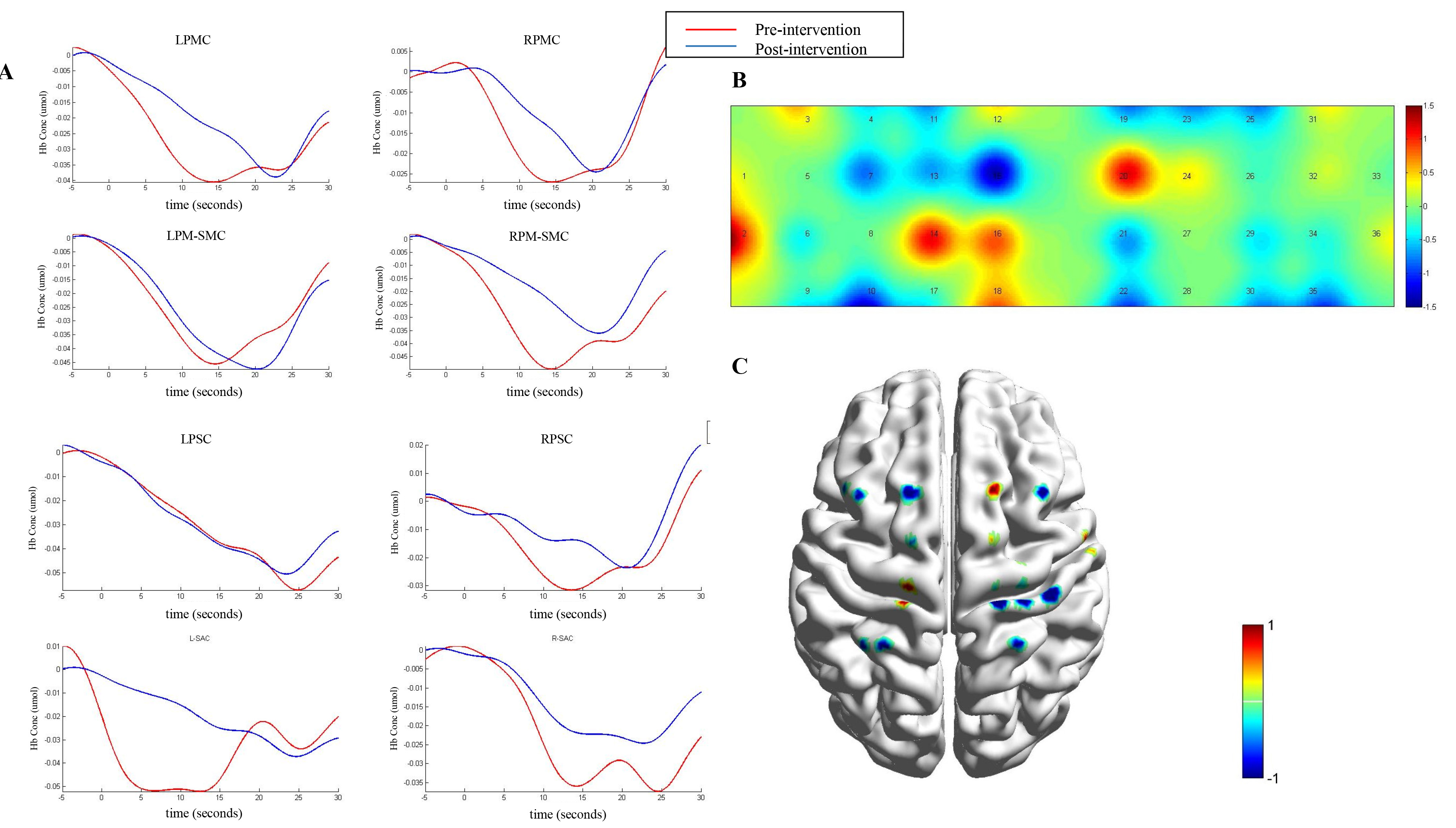

Supplement: Supplementary file 2 — Figure S2 [file CNS-30-e14530-s001.jpg]
